# A Rare Case of Coexisting Breast Cancer and Refractory Acute Myeloid Leukemia

**DOI:** 10.1155/2020/8893185

**Published:** 2020-08-25

**Authors:** L. Ballotta, S. M. Trisolini, A. P. Iori, U. La Rocca, A. Micozzi, G. Gentile, T. De Giacomo, A. Guarini, R. Foà, S. Capria

**Affiliations:** ^1^Hematology, Department of Translational and Precision Medicine, Sapienza University of Rome, Rome, Italy; ^2^Department of Cardiovascular and Thoracic Surgery “Paride Stefanini”, Sapienza University, Policlinico Umberto I, Rome, Italy

## Abstract

The occurrence of acute myeloid leukemia (AML) within six months from a diagnosis of breast cancer (BC) is rarely reported in the literature, and it is associated with a poor prognosis. We report herein the case of a 40-year-old woman referred to our centre affected by BC and simultaneous AML. The patient proved refractory to first line therapy and achieved complete remission (CR) with a clofarabine-based regimen followed by allogeneic stem cell transplantation (ASCT). Both during salvage chemotherapy and after ASCT, the patient presented severe infectious complications ( acute cholecistytis and *Nocardia* pneumonia, respectively) treated with surgery, and currently she is alive in CR for both diseases after 29 months of follow-up. The case highlights the importance of a diagnostic assessment of any unexplained cytopenia in association with solid neoplasia under treatment, underlining the feasibility and priority of a timely treatment of the haematological neoplasm in order to achieve long-term survival.

## 1. Introduction

The prognosis of early-stage breast cancer (BC) in women has radically improved after the introduction of neoadjuvant chemotherapy regimens including anthracyclines and alkylating agents [1]. However, the association between cytotoxic agents and secondary acute myeloid leukemia (AML) has been frequently reported in the literature, and it is included in the 2016 edition of WHO classification as “therapy-related myeloid neoplasms.” While this association is a late complication of chemotherapy and depends on the cumulative dose administered, the synchronous occurrence (within 6 months) of two neoplasms is rarely observed [2]. We hereby report a rare case of coexisting BC and refractory AML focusing on the importance of an early diagnosis of both diseases, and of a shared approach between the hematologist and the oncologist to tailor both the treatment choices and the timing of intervention.

## 2. Case Report

In October 2016, a 40-year-old woman was admitted to another hospital for a diagnosis of BC. One month prior to admission, a routinary mammography had shown the presence of a mass of 15 mm in diameter confirmed by a subsequent MRI scan. She was treated with right quadrantectomy and axillary dissection, and the histopathology provided a diagnosis of grade III invasive poorly differentiated ductal carcinoma. Immunohistochemistry was positive for estrogen and progesterone receptors (75%), with a high Ki67 proliferation index (30%) and mild expression of HER-2 (+1). However, fluorescent in situ hybridization for the HER2 gene was negative (staging pT1N2M0). In November, she started adjuvant chemotherapy regimen with doxorubicin 60 mg/m^2^/day and cyclophosphamide 600 mg/m^2^/day for 4 cycles, followed by maintenance with paclitaxel 80 mg/m^2^ since April 2017 and endocrine therapy with tamoxifen. Peripheral blood cell count at the start of chemotherapy showed leukopenia (white blood cells count 1.0 × 10^9^/L), not further investigated, with mild anemia (hemoglobin 11.6 g/dl) and normal platelets count (168 × 10^9^/L). In June 2017, during breast adjuvant radiotherapy, the patient was persistently pancytopenic (hemoglobin 9.6 g/dl, white blood cells 0.9 × 10^9^/L, and platelets 101 × 10^9^/L). Bone marrow was cellular (+3) and showed 82% of CD34/CD117/CD13+ and CD33/CD7/CD11b/CD14/cMPO blasts of small-medium size, round nucleus, and basophilic cytoplasm with rare granules, consistent with a diagnosis of AML. Cytogenetic analysis showed a normal karyotype (46, XX), and the gene mutation status was wild type for FLT3/ITD and TKD, AML1/ETO, Cbf/MYH11, NPM1, PML-RAR*α*, BCR-ABL1, and MLL. The patient was treated with ICE induction chemotherapy, consisting of a combination of idarubicin 10 mg/m^2^ days 1, 3, and 5, cytarabine 100 mg/m^2^ days 1–7, and etoposide 100 mg/m^2^ days 1–5, but proved refractory. In September 2017, the patient was referred to our centre and underwent a salvage chemotherapy with the G-CLAC schedule: clofarabine 30 mg/m^2^ days 1–5, cytarabine 2000 mg/m^2^ days 1–5, and granulocyte colony stimulating factor (G-CSF) until hemopoietic recovery. On day +13 from chemotherapy, the patient was pancytopenic (hemoglobin 8.5 g/dl, white blood cells 0.750 × 10^9^/L, neutrophils 0.2 × 10^9^/L, and platelets 20 × 10^9^/L) and presented a septic shock, associated with acute abdominal pain with positive Murphy sign and positive blood cultures for *Escherichia coli*, *Staphylococcus haemolyticus,* and *Candida glabrata*. She received antibiotic and antifungal treatment (meropenem, tigecycline, and caspofungin) and supportive care with fluids and inotropic agents. An abdominal ultrasound and an MRI allowed to make a diagnosis of acute cholecistytis and in October 2017 (on day +30 from chemotherapy), after a full hematologic recovery, she underwent a laparoscopic cholecystectomy. The microbiologic examination of pericholecistic liquid revealed a multidrug-resistant *Pseudomonas aeruginosa*. In the same period, she became a KPC-*Klebsiella pneumoniae* rectal carrier. On day +35, the bone marrow aspiration showed a complete remission (CR). For this reason, in November 2017, on day +56 from chemotherapy, she underwent an allogeneic hematopoietic stem cell transplantation (HSCT) from the haploidentical brother after a preparative regimen with TBF (thiotepa 5 mg/kg days −7, −6; fludarabine 50 mg/m^2^ days −5, −4, −3; busulfan 3.2 mg/kg days −5, −4, −3). No major toxicity was observed during the aplastic phase, and the engraftment was obtained on day +17 for PMN and on day +25 for platelets. On day +20, the bone marrow aspirate confirmed a CR with complete donor chimerism, and on day +30, the patient was discharged.

**Figure 1:**
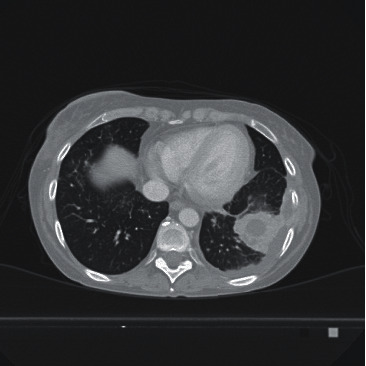
Pulmonary TC scan showing nocardial organized abscess in the left inferior lobe.

Two months after discharge, she was readmitted to the hospital due to an acute left chest pain, fever, and neutropenia (hemoglobin 8.9 g/dl, white blood cells 0.62 × 10^9^/L, neutrophils 0.34 × 10^9^/L, and platelets 21 × 10^9^/L); chest computed tomographic scan showed a basal bilateral pneumonia associated with diffuse pulmonary nodules and bronchial obliteration. A microbiological examination of bronchoalveolar lavage proved the presence of a *Nocardia* spp. Despite targeted antibiotic therapy with high-dose sulfametoxazole-trimethoprim in association with broad-spectrum and specific antibiotics in different timing (amikacin, ceftadizime-avibactam, levoxacin, ceftolozane-tazobactam, tigecycline, imipenem, and linezolid), pulmonary nocardial lesions persisted (and progressed toward) with the formation of a pulmonary organized abscess (Figure 1). Due to the persistence of fever and worsening of clinical conditions, the patient was submitted to a lower pulmonary lobectomy by left lateral thoracotomy; histological examination confirmed the presence of a *Nocardia* spp. She had a good postoperative course with disappearance of fever and complete resolution of the pneumonia.

Currently, the patient is alive in continuous CR for AML and BC, with a follow-up from diagnosis of 29 months and 37 months, respectively. She is in good general conditions with an excellent quality of life.

## 3. Discussion

Herein we describe the case of a young woman presenting with unexplained and persistent cytopenia before and during chemotherapy for BC, due to the coexistence of BC and AML.

The onset of AML within 6 months from a diagnosis of BC is associated with a very poor prognosis with refractoriness to standard therapies and a limited chance to receive an allogeneic HSCT; only 7 cases of a synchronous occurrence of BC and AML have been so far described in the literature (Table 1), and only 3 of them reached a CR for AML. No patient, except the present case, could undergo an allogeneic HSCT and achieved a CR for both diseases. Our patient had a refractory disease for which an allogeneic HSCT currently represents the only recommended therapeutic approach aimed at achieving a long-term disease-free survival [7]. The timing of the different interventions was crucial to bridge this patient to allogeneic HSCT; despite the infectious complications, first of all the acute cholecistytis, she was subjected to laparoscopic cholecystectomy on day +30 from salvage chemotherapy, immediately after a complete hematologic recovery, and to allogeneic HSCT on day +56, after the demonstration of a CR for AML.

In the past years, the use of clofarabine-based regimens as a bridge to transplant has provided encouraging results [8]. Clofarabine is a second-generation purine nucleoside analogue with strong antileukemia activity and an acceptable toxicity profile [9], but its high immunosuppressive effect is well known. It is conceivable that the deep immunosuppression related to clofarabine, associated with posttransplant cyclosporine treatment may have had a role in the occurrence of the *Nocardia* infection, whose incidence in transplanted patients is reported to be about 0.3%, with pulmonary nocardiosis as most frequent manifestation [10]. In the literature, the few cases of *Nocardia* infection described in HSCT patients were treated with antibiotic therapy; no one, with the exception of the hereby reported case, underwent a pulmonary lobectomy. The difficult decision to submit an allogeneic transplanted patient to thoracic surgery was guided by the absence of other therapeutic options.

In conclusion, the occurrence of unexplained cytopenia concomitant to a diagnosis of BC must be closely investigated, and collaboration between the oncologist and hematologist is essential both to reduce the diagnostic delay and to choose the appropriate therapeutic strategy. To our knowledge, our case is the only one described in the literature that is long surviving in complete remission of the two malignancies, characterized by an extremely severe prognosis if it arose simultaneously.

In the molecular era, the coexistence of BC and AML in a young woman could be explained by common pathogenetic mechanisms, and the mutation status of p53 seems to be the most involved: 20–40% of BC patients and 10–20% of AML patients show an acquired mutation of p53 at diagnosis [6]. The presence of p53 mutation is associated with a poor prognosis. In our patient, p53 was not mutated.

Alternative mechanisms involving p53 are described in AML with t(8;21) where the fusion gene AML1-ETO enables the cell to bypass the regulatory effect of p53 [6]. Also abnormalities of chromosome 11 may be involved in AML; chromosome 11 is frequently amplificated in the 11q23 region where the MLL proto-oncogene (myeloid/lymphoid leukemia or mixed-lineage leukemia) is located. In BC, nonrandom abnormalities of chromosome 11 are frequently reported, with involvement of 11p15, 11q13, and 11q23 [6].

In addition, the BRCA1 protein is important for the regulation of cell proliferation, DNA repair, and induction of apoptosis in damaged cells; BRCA1 expression is reduced in about 11–31% of BC, and recent evidences in t-AML have shown a low expression of BRCA1 in AML blasts which could contribute to secondary leukemogenesis, though further studies are necessary [11].

We expect that, in the near future, the new molecular diagnostic techniques as well as the modern biologic therapeutic tools will allow to better understand the molecular pathways possibly involved in the pathogenesis of both diseases, thus opening the way to a combined therapeutic approach.

## Figures and Tables

**Table 1:** Cases of synchronous occurrence of BC and AML in the literature.

Case	Latency	Morphology	Phenotype	Karyotype	Genetics	Outcome
Jacobs and Carey [3]	Simultaneous	NA	NA	NA	NA	NA
Carey [3]	Simultaneous	NA	NA	NA	NA	Died during induction (no autopsy).
Rosner et al. (case 18) [4]	3 months	NA	NA	NA	NA	Died in CR of AML for diabetic coma and gastrointestinal hemorrhage.
Ershler (case 6 and 8) [5]	6 months	NA	NA	45, banding not possible	NA	**RD**. Died in 3 months.
	Simultaneous	M4	NA	NA	NA	Died during induction in 9 days.
Mishra et al. [2]	<1 month	M1	CD13+, CD33+, HLA−DR+, CD7+	46, XX	p53 mutation in both AML and BC	Died from septicemia, DIC, and intracerebral bleeding after reaching a **CR**.
Hu et al. [6]	Simultaneous	M4	HLA-DR+, CD117+, CD34+, CD33+, CD64+, CD11c+, CD13+, CD38+	47, XX, +11	NPM1 and CEBPA mutations	**RD**. Alive in **CR** after 3 lines of chemotherapy. stable BC.
Present case	6 months	—	CD34+, CD117+, CD133+, HLA-DR+, CD4+, CD7+, CD13+, TdT+, CD33+/−	46, XX	Negative	**RD**. Alive in **CR** of AML after allogeneic HSCT. CR of BC.
Our case, data not published	Simultaneous	M2	CD34+/−, CD117+, HLA−DR+, CD13+, CD33+, CD7+	46, XX	MLL self-fusion	**RD**. Died of AML in 13 months.

AML: acute myeloid leukemia; RD: refractory disease; CR: complete remission; NA: not available; BC: breast cancer.

## Data Availability

All data are included within the case study.
